# Validity of an under-mattress sensor for objective sleep measurement in critically ill patients: a prospective observational study

**DOI:** 10.1186/s40560-020-0433-x

**Published:** 2020-02-11

**Authors:** Kanae Nagatomo, Tomoyuki Masuyama, Yusuke Iizuka, Jun Makino, Junji Shiotsuka, Masamitsu Sanui

**Affiliations:** 1 Department of Anesthesiology and Critical Care Medicine, Jichi Medical University Saitama Medical Center, 1-847 Amanuma-cho, Omiya-ku, Saitama-shi, Saitama, 330-8503 Japan; 2 Department of Emergency and Critical Care Medicine, Jichi Medical University Saitama Medical Center, 1-847 Amanuma-cho, Omiya-ku, Saitama-shi, Saitama, 330-8503 Japan; 3 Department of Critical Care Medicine, Yokosuka General Hospital Uwamachi, Uwamachi 2-36, Yokosuka-shi, Kanagawa 238-8567 Japan

**Keywords:** Critically ill patients, Sleep evaluation, Polysomnography, Sleep measurement, Richards–Campbell Sleep Questionnaire

## Abstract

**Background:**

Considering the adverse effects of sleep disturbance in critical care settings, accurate assessment could aid therapy; however, methodological inadequacies mean that no viable option is currently available. Research in healthy population has recently shown that a non-wearable sleep measurement device placed under the mattress of the bed could be beneficial in intensive care settings. Therefore, we aimed to validate this device compared with polysomnography (PSG) and to assess how it related to subjective sleep evaluations.

**Methods:**

This observational study measured the sleep of critically ill adult patients. The primary goal was to validate the Nemuri SCAN (NSCAN; Paramount Bed Co., Ltd., Tokyo, Japan) against the reference standard PSG for 24 h. The secondary goal was to evaluate the association between the objective parameters obtained from NSCAN and PSG and the subjective report data obtained using the Richards–Campbell Sleep Questionnaire (RCSQ) for the nighttime.

**Results:**

Eleven participants were evaluated. The median of the total sleep time scored by PSG was 456.0 (353.0–517.5) min during the nighttime and 305.0 (186.2–542.5) min during the daytime. PSG over 24 h revealed significant decreases in restorative sleep, with excessive daytime sleep, but with a normal quantity of nighttime sleep. The agreement, sensitivity, and specificity rates (with 95% confidence intervals) for the NSCAN compared with PSG were 68.4% (67.9–69.0%), 90.1% (89.7–90.6%), and 38.7% (37.9–39.7%), respectively. The median RCSQ value when subjectively evaluating nighttime sleep was 68.0 (26.3–83.5); this showed no correlation with the NSCAN sleep parameters, despite a positive correlation with the ratio of the stage N2 isolated or combined with restorative sleep in the PSG assessment.

**Conclusions:**

NSCAN had moderate agreement, high sensitivity, and poor specificity in intensive care settings, which is most likely due to its inability to identify immobile wakefulness often observed in critically ill patients or sleep depth. This remains a barrier to its use in the assessment of subjective sleep quality.

**Trial registration:**

This investigation was part of an interventional trial registered with the University Hospital Medical Information Network Individual Clinical Trials Registry (UMIN000026350, http://www.umin.ac.jp/icdr/index-j.html) on March 1, 2017.

## Background

Sleep disturbance, which is a common and negative experience for patients in the intensive care unit (ICU) [1–9], could exacerbate the disease condition and increase the risk of cognitive function deterioration [1–7]. It is also associated with the post-intensive care syndrome that has a significant long-term impact on survivors [10, 11]. The characteristic sleep architectures of this population when measured by polysomnography (PSG) include a reduction in restorative sleep (i.e., rapid eye movement (REM) sleep and slow wave sleep) and severe fragmentation from frequent arousals and awakenings within the normal range of the total sleep time (TST) [1–7], with an abnormality in the day–night sleep cycle [1, 4, 5].

Sleep should be appropriately promoted in critical care settings; however, to date, the factors affecting sleep in the ICU are not completely understood [3, 4, 11]. Furthermore, methodological issues of sleep measurement remain practical barriers [2, 6]. Although PSG is the standard objective method for measuring sleep quality and quantity, its technical difficulty, high cost, and intolerability to patients make it impractical for its routine implementation in the ICU [11, 12]. Although several studies have sought to report alternative methods of sleep monitoring, they have not yet been established [12–15]. The Richards–Campbell Sleep Questionnaire (RCSQ) is one of the simplest methods of subjective evaluation [3, 12, 16] and has been shown to correlate with PSG parameters [17]. However, the reliability of such subjective evaluation is not always assured, particularly in patients with cognitive impairments [3, 6, 12, 18]. Nemuri SCAN (NSCAN; Paramount Bed Co., Ltd., Tokyo, Japan) is a non-wearable sleep monitor placed under a mattress and can automatically identify sleep–wake cycles and whether a patient is in bed by assessing the body motions, respiratory, and heartbeat movements, which has previously been validated in healthy subjects [19].

To date, NSCAN has not been assessed in critically ill patients. Therefore, in this study, we aimed to validate NSCAN against 24-h PSG as a tool for sleep measurement in patients in the ICU and to identify its association with subjective sleep evaluated by the RCSQ. We test a hypothesis that NSCAN is expected to offer the valid precision of detecting patients’ sleep compared with the reference standard PSG for 24 h. The primary outcome variable is patients’ sleep defined by PSG. The primary analysis of the study is agreement, sensitivity, and specificity rates of the patients’ sleep between NSCAN and PSG. We hypothesize that NSCAN is expected to offer an option for sleep evaluation in the ICU.

## Methods

### Study setting and sample

This prospective observational study was part of a larger interventional trial approved by the Institutional Review Board of Jichi Medical University Saitama Medical Center (S17–134). It was conducted in the general 8-bed ICU at Jichi Medical University Saitama Medical Center, a 600-bed tertiary teaching hospital in Saitama, Japan, from March 2017 to October 2017. The staff in the ICU primarily treated perioperative patients (particularly those who have undergone cardiovascular interventions) with severe complications or patients in the hospital requiring mechanical ventilation, continuous renal replacement therapy, or extracorporeal support for acute respiratory failure, septic shock, or other critical illnesses. The ratio of registered nurses to patients was 1:1 or 1:2 during the study depending on the shift patterns and patient needs.

Patients aged ≥ 20 years who were treated in the ICU for at least 72 h were eligible for sleep evaluation for 24 h by PSG, the NSCAN, and the RCSQ. Patients with brain dysfunction, psychiatric disorders, dementia, alcohol or drug abuse, or cardiopulmonary arrest as well as those unable to communicate in Japanese were excluded. Informed consent and written confirmation were obtained from patients or their family before participation. Patients with delirium who were diagnosed in the ICU and who could answer the sleep questions on the measurement day were not excluded because of the reversibility of delirium with daily fluctuation. In our ICU, the nurses routinely assessed the Confusion Assessment Method for the ICU (CAM-ICU) to identify delirium several times a day. Delirium was diagnosed when CAM-ICU was positive even once a day.

### Data collection

Demographic and clinical data were collected from the patients’ electronic medical records, and we calculated the Acute Physiology and Chronic Health Evaluation (APACHE) II score on admission. PSG, the NSCAN, and the RCSQ assessments were then performed.

#### PSG measurement

Patients were monitored for 24 h by PSG (Alice 6 LDx; Philips Respironics, Murrysville, PA, USA) as follows: electroencephalography electrodes were placed according to the international 10–20 system (F4/M1, C4/M1, O2/M1, F3/M2, C3/M2, and O1/M2), electromyography electrodes were placed centrally and on the right and left of the chin, electrooculography was used to monitor the right and left eye movements, and electrocardiography was performed with lead II only. Recording began between 09:00 and 18:00, and trained technicians performed all electrode placements.

PSG recordings were scored manually at 30-s intervals according to the American Association of Sleep Medicine Scoring Manual (version 2.4) [20] by a Registered Polysomnographic Technologist® from an external expert agency who had an experience of 22 years and 4500 patients. All 30-s intervals, called epochs, were judged as each of the following sleep stages: wake (WK), stage N1 (N1), stage N2 (N2), stage N3 (N3), and REM. N1, N2, and N3 are non-REM sleep (NREM), which gradually deepens from N1 to N3. REM occurs next to the deep state of NREM before wake. Each sleep stage was calculated during the day (06:00–20:59) and at night (21:00–05:59). TST was calculated as the total numbers of NREM and REM epochs during the day and at night. Sleep efficiency (SE) was calculated as the daytime TST divided by 900 or the nighttime TST divided by 540. TST and SE represent sleep quantity. The arousal index (ArI), representing sleep fragmentation, was defined as the number of arousals per hour. The frequencies of transition to another sleep stage at night, representing the instability of sleep, were also recorded. Sleep latency and waking after sleep onset were not assessed in this study because of the complexity of defining when nocturnal sleep started or finished in the critical setting, wherein sleep cycles often started before the designated times for lights out (21:00) or after lights on (06:00).

#### NSCAN measurement

An NSCAN was placed under the upper half of each mattress before patient admission and kept in place until discharge from the ICU. The NSCAN can identify when a patient is in or out of the bed based on a pressure sensor. The following parameters were recorded: awakenings, sleep, leaving bed, battery disconnection, body motion, heart rate, and respiratory rate. However, the NSCAN data were collected after the patients were discharged to the ward using a software installed on a personal computer. We only exported the data corresponding to the 24-h period when PSG was performed. Data for each 60-s period were divided into two sets of 30-s data to allow comparison with the 30-s PSG data at the same recording time; for example, when the NSCAN recorded “sleep” for 60 s at time 21:30, we recorded two sleep epochs of 30 s from both 21:30:00 and 21:30:30. The number of nocturnal awakenings was calculated from the NSCAN records as total shifts from sleep to awakening.

#### RCSQ

Patients were asked to fill out the RCSQ to evaluate their nocturnal sleep before or after completion of PSG. The RCSQ assesses sleep depth, latency, frequency of awakenings, latency after awakenings, and sleep quality on 100-mm visual analog scales. The total RCSQ score presents the average value from these five questions, with higher scores indicating better sleep. The original English version of RCSQ was translated into Japanese and validated elsewhere [21]. After receiving approval from the original author, we used a modified Japanese version of the questionnaire with large letters and illustrations. This allowed all critically ill patients, even those who were intubated and required nurse assistance to complete the form, to point on the visual analog scale with their finger.

### Data analysis

The agreement, sensitivity, and specificity rates were calculated from the PSG and NSCAN data measured at the same times. All NREM and REM stages in the PSG recordings were defined as sleep for comparison with the NSCAN data. The NSCAN data indicating sleep or awakenings were validated by the PSG results. The agreement rate represents the ratio of the same judgment, i.e., the epochs that both NSCAN and PSG judged as sleep or wake per total epochs. The sensitivity rate represents the ratio of the epochs scored as sleep by NSCAN per total epochs scored as sleep by PSG. The specificity rate represents the ratio of epochs scored as wake by NSCAN per total epochs scored as wake by PSG. When the NSCAN records indicated either “out-of-bed” or “battery disconnection” statuses, the data for both PSG and NSCAN at that time were excluded from the analysis. Then, we verified the objective sleep parameters from PSG and the NSCAN to identify their association with the subjective RCSQ using Spearman’s rank correlation coefficient. For statistical analyses, we used EZR [22] (R software ver. 3.4.1). Nominal variables are shown as numbers (*n*) with percentage (%), and numerical variables are reported as medians and interquartile ranges of 25–75%. *P* values of < 0.05 were considered statistically significant.

## Results

### Sample characteristics

**Fig. 1:**
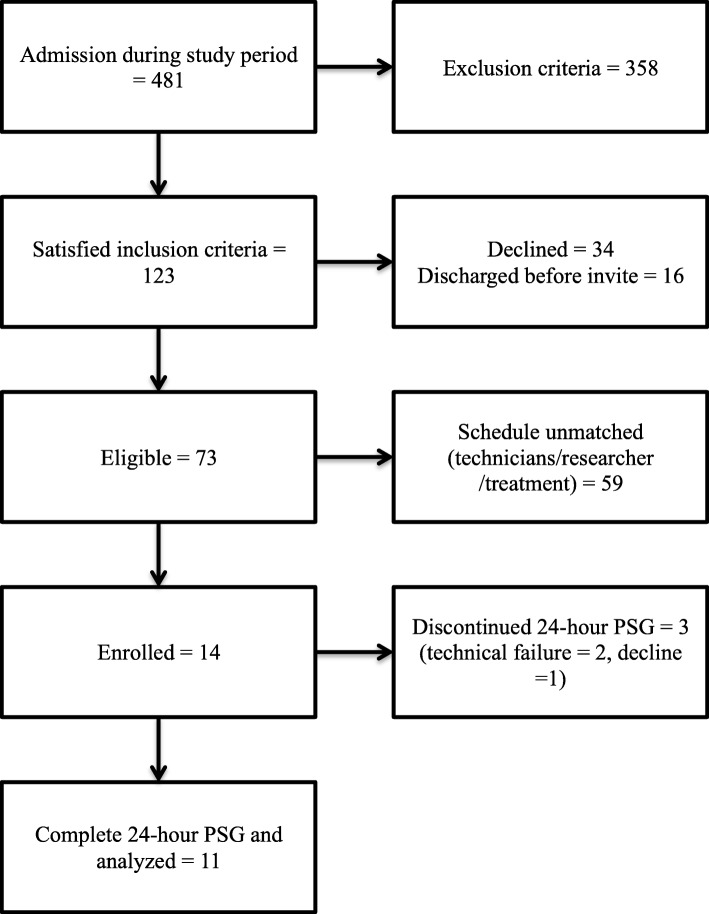
Study flow diagram

In total, 481 patients were admitted to the ICU during the study period, and 123 met the inclusion criteria. Of these, 34 declined PSG monitoring, 16 were discharged before being invited for the study, and 59 did not have treatment schedules compatible with the availability of the technicians or researchers. Finally, 11 of the 14 enrolled patients were evaluated because 1 patient requested removal of PSG after recording began, and 2 other patients had their recordings interrupted by unexpected electrode removal (Fig. 1).

The characteristics of the 11 participants are summarized in Table 1. Most were in the postoperative stage after having undergone cardiovascular surgery (3 emergency cases), and the median APACHE II score (interquartile range) was 18.0 (15.5–22.5). Sleep measurement was performed at a median of 5 days (4.0–5.8 days) from ICU admission. On the day of sleep measurement, 4 patients were intubated on mechanical ventilation, including 1 patient who was sedated with dexmedetomidine, 5 received fentanyl with or without intubation, 1 received ramelteon, and 4 received suvorexant to aid sleep. The Richmond Agitation–Sedation Scale (RASS) was well controlled in all; however, 3 patients were delirious. Neither benzodiazepine nor other sedatives were used.

**Table 1:** Characteristics and clinical data of participants

Total *n* = 11	Number or median	% or IQR
Male, *n*	8	72.7
Age, year	70.0	67.5 to 77.0
APACHE II on admission	18.0	15.5 to 22.5
Medical, *n*	1 (septic shock)	9.1
Perioperative, *n*	10	90.0
Cardiovascular	9	
Hepatic resection	1	
Emergency	3 (all cardiovascular)	
Length of MV, day	2.0	1.5 to 8.5
Length of ICU stay, day	7.0	5.5 to 13.0
28-day survival, *n*	10	90.9
Condition on sleep measurement		
ICU day (day of admission = 1)	5.0	4.0 to 5.8
MV with intubation, *n*	4	36.4
Sedatives, *n*	5	45.5
Dexmedetomidine	1	
Fentanyl	5	
Sleep agent, *n*	5	45.5
Ramelteon	1	
Suvorexant	4	
Maximum RASS		
Daytime (06:00–20:59)	0.0	− 1.0 to 0.0
Nighttime (21:00–05:99)	− 1.0	− 1.0 to − 0.5
Delirium, *n*	3	27.3

*Abbreviations: IQR* 25–75% interquartile range, *MV* mechanical ventilation, *RASS* Richmond Agitation–Sedation Scale

### Association between the sleep monitoring data

The sleep parameters and results obtained by PSG, the NSCAN, and the RCSQ are shown in Table 2. At night (21:00–05:59), the median TST was 456.0 min (353.0–517.0 min), median SE was 84.4% (65.4–95.8%), median REM time was 0.1% (0.0–1.8%), and sleep stages N1, N2, and N3 accounted for 22.2% (14.4–41.4%), 20.5% (19.3–53.5%), and 0.4% (0.0–4.4%), respectively. The median ArI was 14.1 (8.0–20.3), and the median frequency of the sleep stage shift was 8.9 (2.9–11.5). During the day (06:00 to 20:59), the median SE was 33.9% (21.9–60.3%), with 22.2% (13.5–30.0%) in the stage N1 and 8.2% (4.1–19.6%) in the stage N2.

**Table 2:** Results of sleep parameters by the NSCAN and PSG

	Median	Interquartile range
PSG nighttime (21:00–05:59)		
TST (min)	456.0	353.0–517.5
SE (%)	84.4	65.4–95.8
WK (%)	15.6	4.2–34.6
N1 (%)	22.2	14.4–41.4
N2 (%)	20.5	19.3–53.5
N3 (%)	0.4	0.0–4.4
REM (%)	0.1	0.0–1.8
Arousal index (arousals/h)	14.1	8.0–20.3
Frequency of sleep stage shift (/h)	8.9	2.9–11.5
PSG daytime (06:00–20:59)		
TST (min)	305.0	186.2–542.5
SE (%)	33.9	21.9–60.3
WK (%)	66.1	39.7–78.1
N1 (%)	22.0	13.1–30.0
N2 (%)	8.2	4.1–19.6
N3 (%)	0.0	0.0–0.7
REM (%)	0.0	0.0–0.6
NSCAN total recorded epochs (WK+Sleep+Out-of-bed)	30,212	
WK	6233	
Sleep	21,985	
Out-of-bed	1994	
NSCAN battery disconnection	1468	
NSCAN nighttime		
TST (min)	500.0	444.5–525.5
SE (%)	96.3	82.3–98.6
Awakenings	8.0	2.5–11.0
NSCAN daytime		
TST (min)	710.0	517.0–750.5
SE (%)	78.9	65.8–84.3
RCSQ (average of 5 questions)	68.0	26.3–83.5
Sleep depth	62.0	33.0–86.5
Sleep latency	65.0	36.5–81.5
Frequency of awakenings	60.0	17.0–81.5
Sleep latency after awakenings	80.0	21.0–90.0
Sleep quality	68.0	20.0–91.0

The “frequencies of sleep stage shift” indicates the transition number per hour among WK, REM, and NREM

*Abbreviations: Arousal index* arousal numbers per hour, *N1* sleep stage N1, *N2* sleep stage N2, *N3*, sleep stage N3, *NREM* non-rapid eye movement sleep (i.e., N1 + N2 + N3), *NSCAN* Nemuri SCAN, *PSG* polysomnography, *RCSQ* Richards–Campbell Sleep Questionnaire, *REM* rapid eye movement, *TST* total sleep time (N1 + N2 + N3 + REM), *SE* sleep efficiency (TST/540 for nighttime; TST/900 for daytime), *WK* wake

NSCAN recorded a total of 15,106 min, split into 30,212 epochs of 30 s each. Recording was interrupted in one patient because of unplanned battery disconnection, and most out-of-bed time occurred in 1 patient because he preferred being in a seated position without his back against the mattress. At night, the NSCAN recorded a median TST of 500 (444.5–525.5) min, a median SE of 96.3% (82.3–98.6%), and a median of 8.0 (2.5–11.0) nocturnal awakenings; in contrast, the median daytime TST and SE were 710 min (517.0–750.5 min) and 78.9% (65.8–84.3%), respectively.

The validities of the NSCAN and PSG recordings are demonstrated in Table 3. The agreement, sensitivity, and specificity rates (95% confidence intervals) were 68.4% (67.9–69.0%), 90.1% (89.7–90.6%), and 38.8% (37.9–39.7%), respectively. All participants rated their sleep subjectively, giving a median RCSQ value of 68.0 (26.3–83.5). Based on the Spearman’s rank correlation coefficient, the RCSQ showed a significant positive correlation with some PSG parameters: specifically, the nocturnal N2 ratio alone, the stage N2 plus stage N3, and REM sleep (Table 4). No correlation was detected between the RCSQ and the NSCAN parameters.

**Table 3:** Validity of the NSCAN compared with PSG

		95% CI
Agreement, %	68.4	67.9–69.0
Sensitivity, %	90.1	89.7–90.6
Specificity, %	38.8	37.9–39.7
PPV, %	66.8	66.2–67.4
NPV, %	74.2	73.1–75.3
PLR	1.472	1.450–1.495
NLR	0.254	0.241–0.268

*Abbreviations: CI* confidence interval, *NLR* negative likelihood ratio, *NPV* negative predictive value, *NSCAN* Nemuri SCAN, *PLR* positive likelihood ratio, *PPV* positive predictive value, *PSG* polysomnography

**Table 4:** Spearman’s rank correlation coefficient with objective sleep parameters and the RCSQ

	Correlation	*P* value
PSG nighttime		
SE(=TST)	0.000	1.000
N1	− 0.227	0.503
N2	0.727	0.015*
N3	0.191	0.574
REM	0.486	0.130
N1 + N2	0.336	0.313
N2 + N3 + REM	0.700	0.021*
N3 + REM	0.483	0.132
Arousal index	0.000	1.000
Frequency of sleep stage shift	− 0.027	0.946
PSG daytime		
SE(=TST)	0.082	0.818
NSCAN nighttime		
SE(=TST)	0.500	0.121
Awakenings	− 0.540	0.086

The arousal index indicates arousal numbers per hour. The frequencies of sleep stage shift indicate transition numbers per hour among wake, REM, and NREM

*Abbreviations: TST* total sleep time (N1 + N2 + N3 + REM), *SE* sleep efficiency (TST/540 for nighttime, TST/900 for daytime)

*Statistically significant at *p* < 0.05

## Discussion

We aimed to validate the NSCAN as a practical method compared with PSG for assessing sleep in patients in the ICU and to evaluate its association with subjective sleep assessment using the RCSQ. There was a significant decrease in restorative sleep within the normal quantity of nocturnal sleep but with excessive daytime sleep. The NSCAN had high sensitivity and low specificity compared with PSG but did not significantly correlate with the RCSQ results. In contrast, sleep parameters within the stage N2 of PSG correlated with the RCSQ results.

The sleep architectures in our PSG results were similar to those in previous reports of critically ill patients (stage N1, 1–59%; stage N2, 26–74%; stage N3, 0.15–22%; and REM, 1–12%) [7]. The wide ranges for each stage in these previous reports may indicate heterogeneity in the backgrounds, disease severities, and treatments of the critically ill patients. Despite the lack of significant heterogeneity in our small population, similarly wide ranges were evident, especially for the stages N1 and N2. Sleep disruption is characteristic in critical care settings [1, 4, 7], but according to the ArI (1.0–39 per hour) from the previous report [7], it was only mild in our cohort. The instability of nocturnal sleep was only slight based on the frequency of sleep stage shift [23].

Routine implementation of PSG is difficult in large populations or when it is performed for continuous monitoring, as mentioned previously. Therefore, we investigated the NSCAN as an alternative to PSG. For critically ill patients who spend a long time in bed, the NSCAN is easy to use because it is only placed under the mattress and does not include uncomfortable devices that need to be worn. The validity assessment of the NSCAN compared with PSG in this study resulted in agreement, sensitivity, and specificity rates of 68.4%, 90.1%, and 38.7%, respectively. Kogure et al. previously validated the NSCAN against PSG in a healthy population, reporting agreement, sensitivity, and specificity of 92%, 97%, and 34%, respectively [19]. Thus, via both studies, it has been shown that the NSCAN has high sensitivity and low specificity [19]. Given that the NSCAN results are based on patients’ heart rates, respiratory rates, and body motions, it is possible that it does not recognize immobile wakefulness in either healthy individuals or critically ill patients who are less active in bed. However, the difference in the agreement rate suggested that the heart and respiratory rates in the critical setting, likely reflecting the underlying diseases or medical interventions, occurred regardless of sleep or awakenings.

We collected the sleep data for the NSCAN after patients were discharged from the ICU and did not use real-time monitoring. This accounted for the case of unplanned battery disconnection. In another case, in which the patient preferred to remain in a seated position, lack of contact with the mattress showed prolonged out-of-bed times; however, PSG in this case frequently recorded sleep during these out-of-bed times, and the patient sometimes appeared drowsy, even in a seated position. Therefore, it was considered inappropriate to assess sleep or wake based on the out-of-bed data produced by the NSCAN; therefore, these out-of-bed recordings were excluded for all patients from the analysis.

Considering the potential for agreement or discrepancy between objective and subjective sleep evaluation [5, 24, 25], it is important to assess the association between the NSCAN and PSG parameters and their correlation with the RCSQ scores [17]. RCSQ scores range from 46 to 66 [26, 27], but our results exceeded these limits (26.3–83.5), with a higher median value than that reported previously (68). Moreover, the RCSQ results had no correlation with the NSCAN parameters in our study, but they were significantly and positively correlated with some PSG parameters (i.e., stage N2). In a healthy cohort, the ArI and frequency of sleep stage transition are known to relate to subjectively poor sleep quality [23]. Most of the sleep time in our study was in the stage N1 or N2, meaning that sleep could have been subjectively satisfactory despite a lack of the stage N3 and REM sleep. Further, sleep continuity did not appear to be severely disrupted. In contrast, the NSCAN simply assessed sleep or awakening, irrespective of sleep depth. Given the low specificity of the NSCAN, it might overestimate sleep, especially when sleep disruption is mild. These particularities of the NSCAN could have led to a loss of correlation with the RCSQ results.

Although all the participants completed the RCSQ, the scores varied widely, and some participants were visibly tired while providing responses. Among the participants, sedative medications were used (e.g., low-dose fentanyl and dexmedetomidine), which maintained a target RASS from − 1 to 0, and three patients were delirious. For patients with poor alertness, even the five simple questions in the RCSQ could have been annoying, indicating difficulty in self-evaluation even though they could communicate. The feasibility of using this self-assessment method should be considered with care in patients who have any altered state of consciousness [3, 6, 12, 18].

### Limitations

Our study has several limitations. First, because of the single-center design and small study population mainly including patients who had undergone cardiovascular surgery and were in the perioperative period, our data cannot be generalized. Furthermore, the poor enrollment to eligibility rate should be considered owing to the difficult situation for PSG measurements, such as holidays or weekends and patients’ schedule of moving to another bed without NSCAN inside the ICU or to another unit. Although our PSG data were compatible with the sleep patterns reported previously for critically ill patients, heterogeneity of the research population is desirable. Second, the reliability of the RCSQ in patients with altered consciousness (e.g., delirium or sedative/opiate use) could not be verified. In general, multiple conscious states might be included in such conditions; however, target sedation was well controlled and delirium was diagnosed even with one positive CAM-ICU in our study. All the enrollments, including patients with delirium, were able to communicate; therefore, the altered consciousness was suggested as simple as poor attention or concentration with respect to difficulty in providing answers to the RCSQ. Given that delirium is common in the ICU and is associated with sleep disturbance, the evaluation from such altered consciousness should be taken into consideration. Although subjective sleep quality seemed to require achieving at least the stage N2 on PSG, the factors contributing to subjective sleep quality were unclear. Third, the atypical PSG features in critical care settings are of concern [24, 25]. Pathologic wakefulness is characterized by the appearance of slow waves (representing stage N3) with a wakeful appearance and indicates encephalopathy [5, 7]. In this study, only one patient in the postoperative period (resection of hepatocellular carcinoma) had these features in the subjective sleep evaluation, although the effect was small on the overall analysis. Other factors, such as the use of sedatives, opiates, or mechanical ventilation could also affect PSG features [1–3, 5, 7, 25], adding to the analytical complexity of PSG in the ICU [25]. Thus, although PSG is the gold standard for sleep assessments, its accuracy is still unclear in this setting [28]. Adding to the third limitation, it might be considered in cases of out-of-bed patients. One of our patients who preferred a seated position in a drowsy appearance was judged as out-of-bed by NSCAN for multiple moments; thus, the out-of-bed data were excluded from the analysis irrespective of the state that PSG described. Furthermore, in ordinary settings, we often encounter the situation where patients are out of their ICU beds due to experiments, hemodialysis, surgical operations, or patients’ posture. In such cases, the condition of sleep or wake depends on a case-by-case basis, similar to our patient. It might be possible to estimate sleep or wake from the condition; nevertheless, it is inappropriate to include the estimated data for the analysis.

Despite these limitations, this is the first report to have validated the NSCAN data against PSG recordings over 24 h in a critical care setting. It is also the first report comparing a non-wearable device for objective sleep assessment against the PSG and RCSQ data. Further investigation is now required to confirm our findings. In particular, this research should include participants with clinical heterogeneity and from multiple centers, and there should be efforts to delineate the role of factors that affect consciousness.

## Conclusions

The NSCAN showed moderate agreement, high sensitivity, and low specificity in critical care settings when compared with PSG as the reference standard. Although subjective sleep evaluation by RCSQ positively correlated with the parameters measured by PSG, there was no correlation with sleep parameters measured by the NSCAN. Further investigation is required to identify the role of NSCAN in critically ill patients.

## Data Availability

The datasets used for the current study are not publicly available but will be made available by the corresponding author on reasonable request.

## Abbreviations

ArI: Arousal index
ICU: Intensive care unit
NSCAN: Nemuri SCAN
PSG: Polysomnography
RASS: Richmond Agitation–Sedation Scale
RCSQ: Richards–Campbell Sleep Questionnaire
REM: Rapid eye movement
SE: Sleep efficiency
TST: Total sleep time
